# Identification of diagnostic peptide regions that distinguish Zika virus from related mosquito-borne Flaviviruses

**DOI:** 10.1371/journal.pone.0178199

**Published:** 2017-05-31

**Authors:** Alexandra J. Lee, Roshni Bhattacharya, Richard H. Scheuermann, Brett E. Pickett

**Affiliations:** 1 J. Craig Venter Institute, La Jolla, California, United States of America; 2 Biological and Medical Informatics, San Diego State University, San Diego, California, United States of America; 3 Department of Pathology, University of California, San Diego, California, United States of America; 4 La Jolla Institute for Allergy and Immunology, La Jolla, California, United States of America; University of Texas Medical Branch at Galveston, UNITED STATES

## Abstract

Zika virus (ZIKV) is a member of the *Flavivirus* genus of positive-sense single-stranded RNA viruses, which includes Dengue, West Nile, Yellow Fever, and other mosquito-borne arboviruses. Infection by ZIKV can be difficult to distinguish from infection by other mosquito-borne Flaviviruses due to high sequence similarity, serum antibody cross-reactivity, and virus co-circulation in endemic areas. Indeed, existing serological methods are not able to consistently differentiate ZIKV from other Flaviviruses, which makes it extremely difficult to accurately calculate the incidence rate of Zika-associated Guillain-Barre in adults, microcephaly in newborns, or asymptomatic infections within a geographical area. In order to identify Zika-specific peptide regions that could be used as serology reagents, we have applied comparative genomics and protein structure analyses to identify amino acid residues that distinguish each of 10 *Flavivirus* species and subtypes from each other by calculating the specificity, sensitivity, and surface exposure of each residue in relevant target proteins. For ZIKV we identified 104 and 116 15-mer peptides in the E glycoprotein and NS1 non-structural protein, respectively, that contain multiple diagnostic sites and are located in surface-exposed regions in the tertiary protein structure. These sensitive, specific, and surface-exposed peptide regions should serve as useful reagents for seroprevalence studies to better distinguish between prior infections with any of these mosquito-borne Flaviviruses. The development of better detection methods and diagnostic tools will enable clinicians and public health workers to more accurately estimate the true incidence rate of asymptomatic infections, neurological syndromes, and birth defects associated with ZIKV infection.

## Introduction

Zika virus (ZIKV) belongs to the *Flavivirus* genus in the *Flaviviridae* family of positive-sense, single-stranded RNA viruses. This genus also includes Dengue (DENV), West Nile (WNV), Yellow Fever (YFV), and other arthropod-borne viruses. The ~10.8 kb genome produces a single polyprotein that is co- and post-translationally processed into 10 mature proteins by host and virus-encoded proteases. ZIKV can be classified into three phylogenetic lineages, East African, West African and Asian, and is transmitted primarily through the bite of an infected *Aedes* mosquito, with evidence also supporting sexual transmission [1–4]. ZIKV had previously only been detected in sporadic outbreaks in Africa, Southeast Asia and the Pacific Islands [5], until early 2015 when it emerged in eastern Brazil [6, 7]. Since then, the Asian lineage has rapidly spread throughout South and Central America with limited travel-related cases reported in Europe and Asia as well as autochthonous transmission in the Southeastern United States. Historically, ZIKV infections were thought to be associated with mild or asymptomatic viral disease. However, a relatively high frequency of neurological syndromes (e.g. Guillain-Barre) and birth defects (e.g. microcephaly) associated with the recent ZIKV outbreak have contributed to the WHO declaring ZIKV a global public health emergency [8–11].

Diagnostic identification of infection by these viruses currently requires detecting viral genetic material in blood samples taken from patients during acute infection [12]. Unfortunately, nucleotide-based methods are not always plausible due to the required laboratory infrastructure and a limited window of detection when viral particles are circulating [13]. In addition, accurately detecting whole Flavivirus proteins from patient samples taken during acute infection has had limited success due to broad cross-reactivity of existing serological reagents [12, 14–20].

Precisely calculating the incidence and prevalence rates for ZIKV is extremely difficult due to: co-circulation of other mosquito-borne Flaviviruses in the same geographical area [21], their similar clinical signs and symptoms [14], and under-reporting of asymptomatic infections [22]. The detection of anti-viral antibodies in patient sera has been used successfully in the past to improve incidence and prevalence estimates for other viruses such as Human Immunodeficiency Virus and Hepatitis C virus [23, 24]; however, this approach is dependent on the sensitivity and specificity of the antibody-binding reagents used [25, 26]. In this study, we performed a computational analysis of Flavivirus E and NS1 proteins across 10 species and subtypes to identify individual amino acid residues and peptide regions that are unique to each mosquito-borne Flavivirus. The sensitive and specific peptide regions that were identified through this analysis will be used to develop improved serological diagnostic methods for detecting past infection with these viral pathogens.

## Materials and methods

### Sequence retrieval and filtering

Sequence data for the E and NS1 proteins from Dengue (DENV1-4), Ilheus (ILHV), Japanese encephalitis (JEV), St. Louis encephalitis (SLEV), West nile (WNV), Yellow fever (YFV), and Zika (ZIKV) viruses were retrieved from the Virus Pathogen Database and Analysis Resource (ViPR, www.viprbrc.org) in March 2017 [27]. Sequences for each taxon were filtered to remove duplicate, incomplete, and poor-quality sequences to minimize the introduction of bias and improve downstream analyses. In order to ensure an accurate multiple sequence alignment across the different taxa using MAFFT [28], we removed any E or NS1 sequence that was not at least 75% complete, excluding ILHV since there was an insufficient number of sequences for this species.

### Regions with high sensitivity and specificity

Sequences were assigned into 2 groups–species X (e.g. ZIKV) versus non-species X (e.g. all Flaviviruses other than ZIKV). A custom script was used to calculate the sensitivity and specificity of all aligned residues using the predominant amino acid residue found in the single taxon group as the diagnostic residue. Residues having an average sensitivity and specificity of at least 98% were labeled as *diagnostic sites*.

A sliding window with a window size of 15 amino acid positions and a step size of 1 position was used to identify regions containing at least 3 residues that exceeded this sensitivity/specificity threshold. (S2 and S3 Tables)

### Surface exposure calculation & protein structure analysis

Solvent-accessible surface areas for the ZIKV E and DENV2 NS1 protein structures were calculated using PDB files 5IRE and 4O6B respectively in the Chimera tool suite [29]. The Chimera tool calculates the surface accessibility for each protein chain individually. Therefore, the surface accessibility scores for the E protein were manually adjusted so that the residues within the transmembrane region, as annotated by UniProt, were set to 0; the surface accessibility scores of the NS1 protein were not adjusted. The relative surface accessibility (RSA) was calculated by normalizing the surface area at each position by the surface area of their respective amino acid residue [30]. Using the same sliding window as before, all 15-mers that contained 6 or more residues with relative surface accessibility values greater than the average for only hydrophilic residues (0.321 for E and 0.281 for NS1) were identified and used for further analysis.

The tools within SWISS-MODEL were used to identify the ideal three-dimensional structures for each of the 10 viral taxa [31, 32]. Specifically, template identification for the 10 Flavivirus taxa was performed using BLAST and HHblits with thresholds set at greater than 80% coverage, 40% sequence similarity, and 40% sequence identify [33–36]. PDB structures for the mature E protein crystal structures of DENV2 (3J2P) and ZIKV (5IRE) were then used for modeling (www.rcsb.org) [37–39]. Three-dimensional structure predictions for the remaining taxa were predicted with Modeller and ProMod onto either one of the existing structures as templates [40, 41]. Model quality was assessed using QMEAN and GMQE values [31, 42], prior to structural alignment of each taxa in Chimera.

### Overlapping immune epitopes

The cumulative number of non-redundant amino acid positions in the surface diagnostic peptide regions (i.e. 15-mer diagnostic peptide regions that contained at least 6 surface-exposed residues and at least 3 sensitive and specific residues) were iteratively calculated for each of the 10 Flavivirus taxa. The cumulative number of non-redundant positions located in published human B-cell epitopes were also calculated for the ten Flavivirus taxa, using data retrieved from ViPR and the Immune Epitope Database (www.iedb.org) [43]. A percentage representing the number of sites that overlapped between surface diagnostic peptide regions and B-cell epitopes was then calculated.

### Code availability

The scripts, code, input files, and workflow that were used in this work are publicly available at: www.github.com/ajlee21/ZIKV_diagnostic-.

## Results

The principle aim of this work was to identify individual peptide regions that uniquely distinguish the E and NS1 proteins for each of 10 different mosquito-borne Flaviviruses species and subtypes. To be as comprehensive as possible, we included sequence records from the following Flavivirus species/subtypes in our analysis: Dengue 1 (DENV1), Dengue 2 (DENV2), Dengue 3 (DENV3), Dengue 4 (DENV), Ilheus (ILHV), Japanese encephalitis (JEV), St. Louis encephalitis (SLEV), West Nile (WNV), Yellow fever (YFV), and Zika (ZIKV) viruses. We specifically chose these Flaviviruses based on: their ability to infect humans, their use of a mosquito vector, their phylogenetic relatedness, the number of publicly available sequence records, and expected challenges associated with serological cross-reactivity. The E and NS1 proteins were chosen because they have been found to be the primary extracellular antigens that elicit host adaptive immune responses during viral infection and have been shown previously to be the targets of humoral immune responses in humans [44, 45]. Peptides that are sensitive and specific for these viral species, which are predicted to be exposed to anti-viral antibodies, could therefore be used as serodiagnostics.

### Diagnostic sites

We began by collecting all of the available E and NS1 sequences from the 10 relevant species and subtypes in the *Flavivirus* genus (Tables 1 and 2) based on the criteria described in the Methods section.

**Table 1:** E protein sequence counts by species/subtype.

Species Name	Sequence Count	Adjusted Sequence Count^a^
Dengue Virus 1(DENV1)	4,616	4,321
Dengue Virus 2(DENV2)	4,169	3,614
Dengue Virus 3(DENV3)	2,330	2,038
Dengue Virus 4(DENV4)	1,483	1,372
Ilheus Virus (ILHV)	4	4
Japanese Encephalitis Virus (JEV)	1,440	1,243
St. Louis Encephalitis Virus (SLEV)	241	189
West Nile Virus (WNV)	3,154	3,044
Yellow Fever Virus (YFV)	382	169
Zika Virus (ZIKV)	347	264

^a^ Shows the number of sequences that remained in the analysis after filtering.

**Table 2:** NS1 protein sequence counts by species/subtype.

Species Name	Sequence Count	Adjusted Sequence Count^a^
Dengue Virus 1(DENV1)	1,822	1,822
Dengue Virus 2(DENV2)	1,529	1,527
Dengue Virus 3(DENV3)	997	997
Dengue Virus 4(DENV4)	364	228
Ilheus Virus (ILHV)	6	6
Japanese Encephalitis Virus (JEV)	312	300
St. Louis Encephalitis Virus (SLEV)	129	41
West Nile Virus (WNV)	1,894	1,893
Yellow Fever Virus (YFV)	89	79
Zika Virus (ZIKV)	251	232

^a^ Shows the number of sequences that remained in the analysis after filtering.

In order to determine which amino acid residues uniquely distinguish between each taxon compared to all the other taxa, we calculated the sensitivity (i.e. the conservation of an amino acid residue in the taxon in question) and specificity (i.e. the uniqueness of an amino acid residue for the taxon in question) at each position, using the methods outlined above. To reduce the number of false positive results, we applied a stringent cutoff by retaining only those positions with average sensitivity and specificity values exceeding 98% (Tables 3 and 4). For the ZIKV E protein, there were 86 residue positions that met our stringent criteria. One of these diagnostic sites, which was located at aligned position 205 (unaligned position 197 in the E protein from strain MR 766, GenBank accession AY632535), contained a Y residue in 261 ZIKV sequences and a mix of 4 F, 169 I, 11326 L, 2 M, 6 S, 4480 V, 1 X in the 9 remaining taxa. By applying this strict set of criteria, the list of residues in the final output were considered to be sufficiently unique for consideration in the development of diagnostics or detection methods for these viruses.

**Table 3:** Summary statistics for the E protein.

Group 1	Count 1	Group 2	Count 2	Number of Diagnostic Sites^a^	Diagnostic Peptides ^b^	Surface-exposed Diagnostic Peptides ^c^
Dengue Virus 1(DENV1)	997	All Other	5305	33	55	41
Dengue Virus 2(DENV2)	1,000	All Other	5302	61	137	80
Dengue Virus 3(DENV3)	1,000	All Other	5302	38	74	50
Dengue Virus 4(DENV4)	897	All Other	5405	72	166	91
Ilheus Virus (ILHV)	4	All Other	6298	41	78	35
Japanese Encephalitis Virus (JEV)	1,000	All Other	5302	54	121	73
St. Louis Encephalitis Virus (SLEV)	178	All Other	6124	57	149	75
West Nile Virus (WNV)	993	All Other	5309	54	115	69
Yellow Fever Virus (YFV)	162	All Other	6140	156	432	223
Zika Virus (ZIKV)	71	All Other	6231	86	228	104

^a^ Candidate diagnostics sites that showed average sensitivity/specificity above 98%.

^**b**^The number of 15-mer peptides that contain at least 3 candidate diagnostic sites for each Flavivirus taxon.

^**c**^The number of 15-mer peptides for each Flavivirus taxon that contain at least 3 candidate diagnostic sites and at least 6 surface-exposed residues.

**Table 4:** Summary statistics for the NS1 protein.

Group 1	Count 1	Group 2	Count 2	Number of Diagnostic Sites^a^	Diagnostic Peptides ^b^	Surface-exposed Diagnostic Peptides ^c^
Dengue Virus 1(DENV1)	1,000	All Other	3499	28	31	16
Dengue Virus 2(DENV2)	1,000	All Other	3499	23	46	23
Dengue Virus 3(DENV3)	951	All Other	3548	22	55	24
Dengue Virus 4(DENV4)	185	All Other	4314	36	65	33
Ilheus Virus (ILHV)	6	All Other	4493	21	51	26
Japanese Encephalitis Virus (JEV)	227	All Other	4272	41	95	44
St. Louis Encephalitis Virus (SLEV)	36	All Other	4463	50	127	57
West Nile Virus (WNV)	988	All Other	3511	32	57	38
Yellow Fever Virus (YFV)	72	All Other	4427	114	308	133
Zika Virus (ZIKV)	34	All Other	4465	80	235	116

^a^ Candidate diagnostics sites that showed average sensitivity/specificity above 98%.

^**b**^The number of 15-mer peptides that contain at least 3 candidate diagnostic sites for each Flavivirus taxon.

^**c**^The number of 15-mer peptides for each Flavivirus taxon that contain at least 3 candidate diagnostic sites and at least 6 surface-exposed residues.

These individual diagnostic residues simultaneously represent evolutionary divergence between the shared common ancestor of these Flavivirus species/subtypes, and evolutionary conservation within any individual Flavivirus species/subtype. Given the current need to develop specific and sensitive diagnostics capable of distinguishing between these mosquito-borne viruses, the diagnostic value of a peptide region increases when it contains multiple nearby unique residues.

### Diagnostic peptide regions

**Fig 1:**
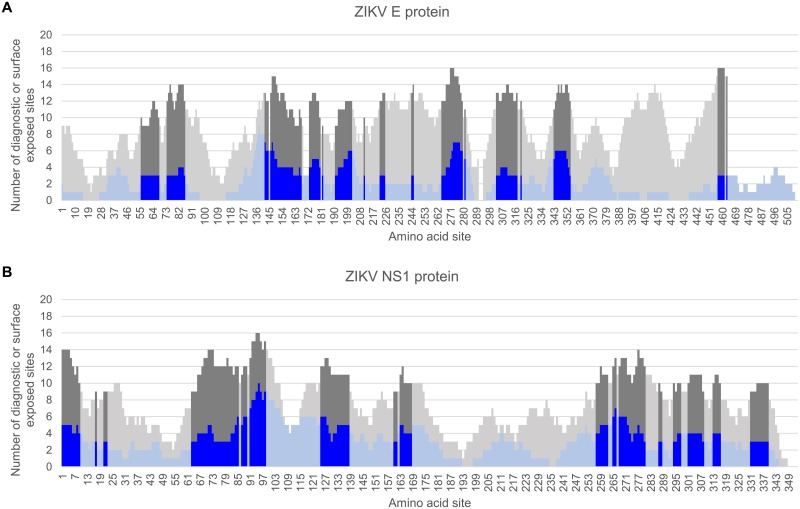
Identification of diagnostic peptide regions for ZIKV. Stacked bar chart of diagnostic sites having high sensitivity and specificity within a sliding window (window size of 15, step size of 1) for the ZIKV E (A) and ZIKV NS1 (B) proteins. Y-axis indicates the number of diagnostic residues (blue bars) or surface exposed residues (gray bars) in the 15-mer peptide starting at the protein amino acid position indicated on the x-axis. Surface-exposed diagnostic peptide regions containing at least 3 diagnostic sites and 6 solvent-accessible residues are represented with darker shading.

**Fig 2:**
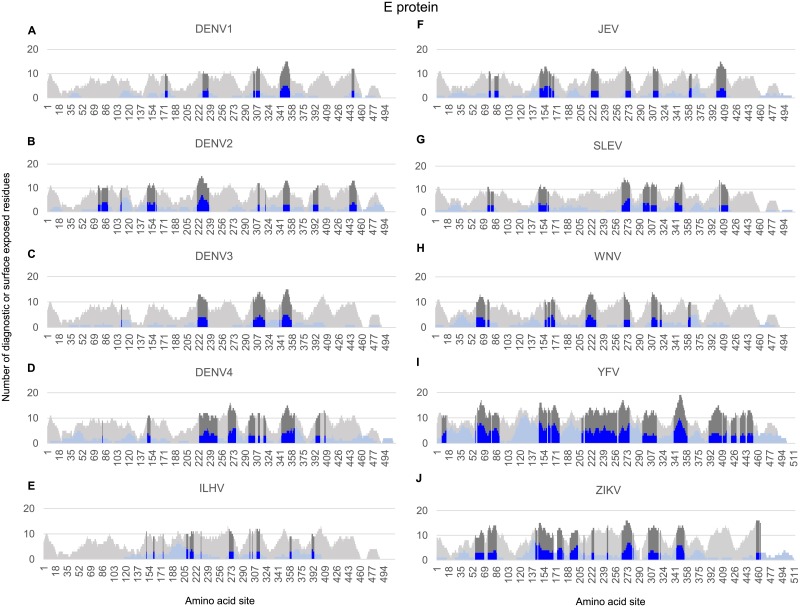
Identification of diagnostic peptide regions in the E glycoprotein for all Flavivirus taxa infecting humans. Stacked bar chart of candidate diagnostic sites (i.e. amino acid positions that were found to have high sensitivity and specificity for each Flavivirus taxon) within a sliding window (window size of 15, step size of 1) for the E protein sequences in each of the Flavivirus taxa. Y-axis indicates the number of diagnostic residues (blue bars) or surface exposed residues (gray bars) in the 15-mer peptide starting at the protein amino acid position indicated on the x-axis. Surface-exposed diagnostic peptide regions containing at least 3 diagnostic sites and 6 solvent-accessible residues are represented with darker shading.

**Fig 3:**
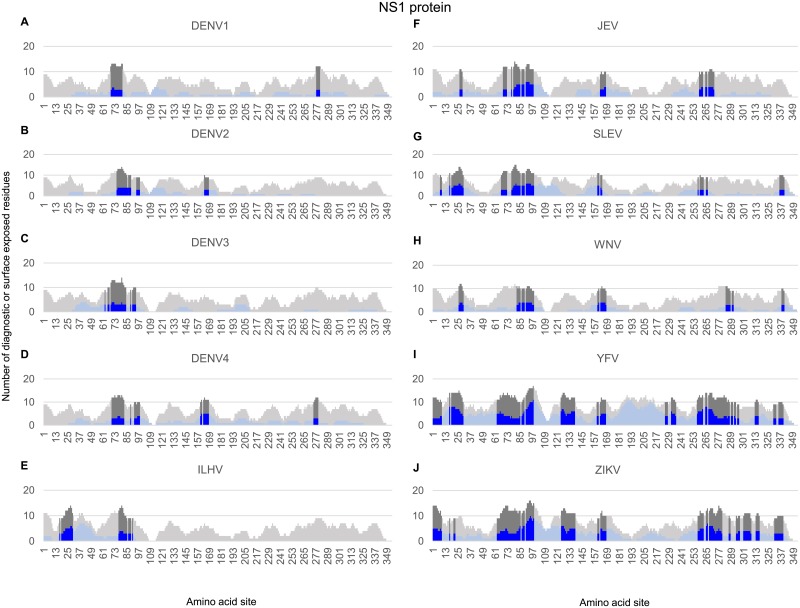
Identification of diagnostic peptide regions in the NS1 non-structural protein for all Flavivirus taxa infecting humans. Stacked bar chart of candidate diagnostic sites (i.e. amino acid positions that were found to have a high degree of sensitivity and specificity for each Flavivirus taxon) within a sliding window (window size of 15, step size of 1) for the NS1 protein sequences in each of the Flavivirus taxa. Y-axis indicates the number of diagnostic residues (blue bars) or surface exposed residues (gray bars) in the 15-mer peptide starting at the protein amino acid position indicated on the x-axis. Surface-exposed diagnostic peptide regions containing at least 3 diagnostic sites and 6 solvent-accessible residues are represented with darker shading.

Since one of the primary goals of this study was to identify protein regions that would be predicted to have high sensitivity and specificity for binding by antiviral serum antibodies, we wanted to identify extended linear peptide regions that included multiple diagnostic residues. We identified these *diagnostic peptide regions* by using a sliding window approach, counting the number of diagnostic sites located within a 15 amino acid window. For the ZIKV E protein we identified 102 15-mers that contained 3 diagnostic residues, and 50, 29, 37, 4, and 6 15-mers that contained 4, 5, 6, 7, and 8 or more such residues, respectively (Fig 1). Figs 2 and 3 show the counts for sliding windows across the length of the E and NS1 proteins for all 10 Flavivirus taxa. We selected a cutoff of 3 diagnostic residues in a 15-mer region as a definition of a candidate diagnostic peptide region since this number of amino acid changes within a B-cell epitope are predicted to be sufficient to adversely affect antibody binding affinity. Using this sliding window approach and these selection criteria we identified 228 diagnostic peptide regions in the ZIKV E protein and 235 peptides in the NS1 protein. The number of diagnostic peptide regions in the E and NS1 proteins for all 10 Flavivirus taxa are listed in Tables 3 and 4, respectively.

**Fig 4:**
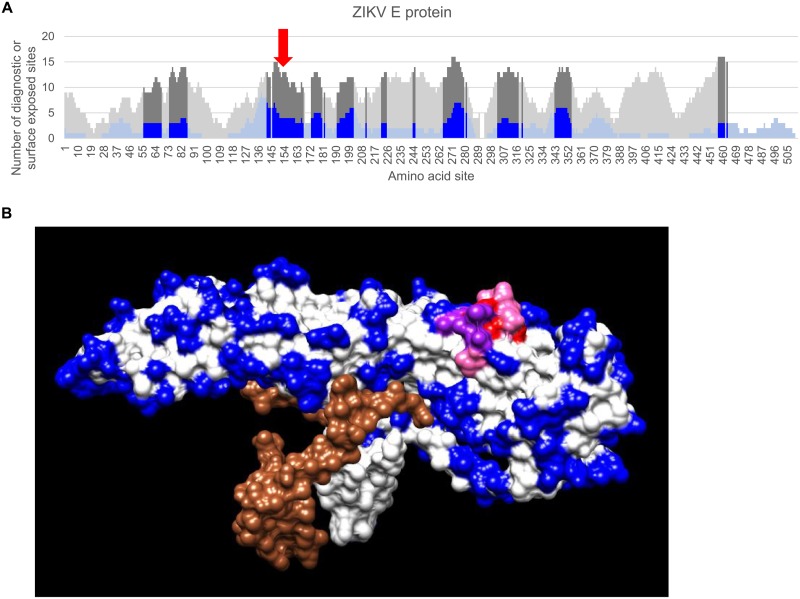
Location of a selected surface-exposed diagnostic peptide region in the E glycoprotein structure. (A) Plot providing higher resolution of the ZIKV E results shown in Fig 1 with a red arrow indicating the surface diagnostic peptide highlighted in the lower panel. (B) Surface view of the structure for the ZIKV E:M heterodimer (PDB: 5IRE) is shown. The M chain is colored brown, the E chain is colored white, the selected 15-mer is colored red, residues that are surface exposed are colored blue, residues that overlap between the 15-mer peptide and surface exposed residues are colored purple, candidate diagnostic residues within the 15-mer that overlap with surface exposed residues are colored pink.

**Fig 5:**
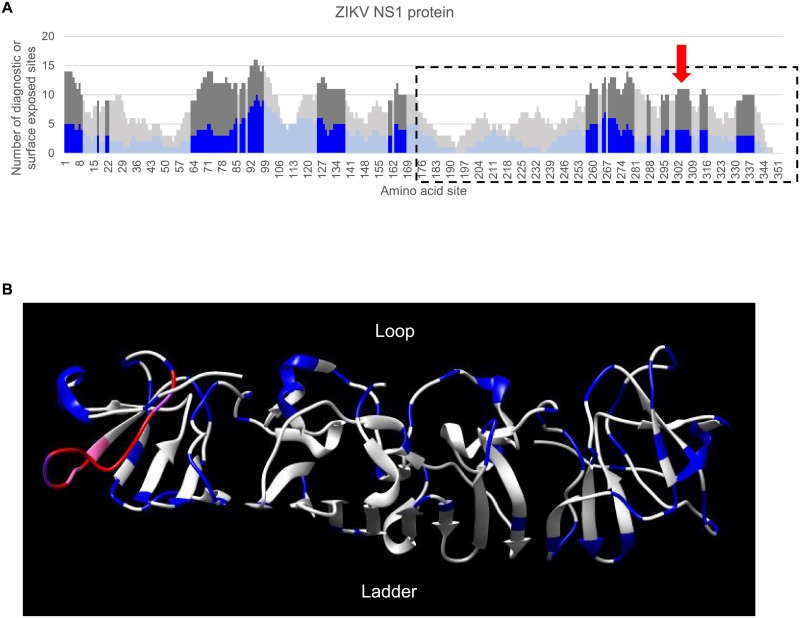
Location of a selected surface-exposed diagnostic peptide region in the NS1 nonstructural protein structure. (A) Plot providing higher resolution of the ZIKV NS1 results in Fig 1. The crystallized portion of this protein is outlined by a black dotted line and the red arrow indicates the diagnostic peptide region highlighted in the lower panel. (B) Ribbon view of the structure for the ZIKV NS1 C terminus (PDB: 5IY3). The loop region faces outward and is completely exposed on the surface while the ladder region faces inward. The NS1 C-terminus is colored white, the selected 15-mer with 4 diagnostics residues is colored red, residues that are surface exposed are colored blue, residues that overlap between the 15-mer peptide and surface exposed residues are colored purple, candidate diagnostic residues that overlap with surface exposed residues colored pink.

In order to further refine the candidate list of diagnostic peptide regions, we next identified regions that are predicted to be exposed on the surface of the protein and would therefore be accessible for binding by host anti-viral antibodies. To enable this analysis, the diagnostic sites that were obtained through multiple sequence alignment and both sensitivity and specificity analysis were then merged with the solvent accessibility values by manually mapping the positions in the global alignment to the PDB amino acid numbers for either the ZIKV E protein or the DENV2 NS1 protein structures. We then calculated the accessible surface area for each amino acid residue from the relevant 3D protein structures. Linear regions in each protein that had at least 6 amino acid residues within a 15 amino acid window with relative surface accessibility values exceeding the average exposed area of hydrophilic residues were selected as *surface-exposed diagnostic peptide regions* (S4 and S5 Tables). A cutoff value of 6 surface-exposed residues was specifically chosen for two reasons: it is the average length of reported DENV2 epitopes, and it is a slightly more conservative value than the average of 5 residues previously reported to contribute to antibody binding [46, 47]. Visual inspection of a selected diagnostic peptide region, containing at least 3 diagnostic residues, and at least 6 surface-accessible residues, on the 3D protein structures of the ZIKV E (Fig 4) and DENV 2 NS1 (Fig 5) proteins confirmed the solvent accessibility of the diagnostic residues within the diagnostic peptide region.

**Fig 6:**
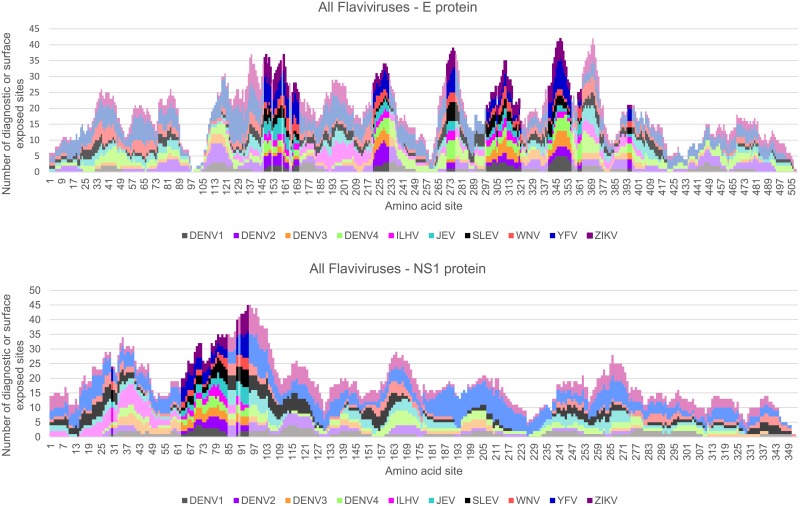
Comparison of the diagnostic peptide regions for all Flavivirus taxa infecting humans. Stacked bar chart of candidate diagnostic peptide sites across the E (upper panel) or NS1 (lower panel) for all 10 of the mosquito-borne Flavivirus species/subtypes evaluated in this study. Areas with darker shading indicate regions where all 10 taxa share a 15-mer peptide that is surface exposed and contains at least one diagnostic site.

We then determined the extent to which the surface diagnostic peptide regions overlapped between the 10 taxa being analyzed. Interestingly, we identified 10 contiguous regions in the E protein and 5 regions in the NS1 protein that contained at least 1 diagnostic and exposed site across all taxa (Fig 6). These regions contain one or more diagnostic sites that significantly differ between individual taxa, which implies that they are conserved within a given species/subtype yet divergent between each species/subtype. Whether these regions are valuable for viral cross-reactivity, neutralization, or diagnostics is still unknown and requires additional investigation.

To validate that these computationally predicted diagnostic peptide regions are likely to serve as targets for serum antibodies, we determined the percentage of residues within surface-exposed diagnostic 15-mer peptides for each of the Flavivirus taxa that overlapped with experimentally-determined human B-cell epitopes across all 10 taxa (Table 5). Our results revealed a mean and median percent overlap of 77.2% (range 68.9% to 86.1%) across all of the ten taxa. These values show the ability of our analytical workflow to produce a set of virus-specific, surface-exposed peptides that are capable of distinguishing between mosquito-borne Flaviviruses and are likely to be recognized by serum antibodies.

**Table 5:** Percentage of amino acid positions within surface diagnostic E protein peptides that overlap with known human B-cell Flavivirus epitopes.

Species	Number of sites within surface diagnostic peptides	Number of sites within surface diagnostic peptides AND B-cell epitopes	Percentage of sites
**DENV1**	112	84	75.0%
**DENV2**	202	174	86.1%
**DENV3**	108	83	76.9%
**DENV4**	204	158	77.5%
**ILHV**	160	126	78.8%
**JEV**	178	141	79.2%
**SLEV**	164	113	68.9%
**WNV**	161	121	75.2%
**YFV**	344	279	81.1%
**ZIKV**	230	169	73.5%

### Protein structure modeling

**Fig 7:**
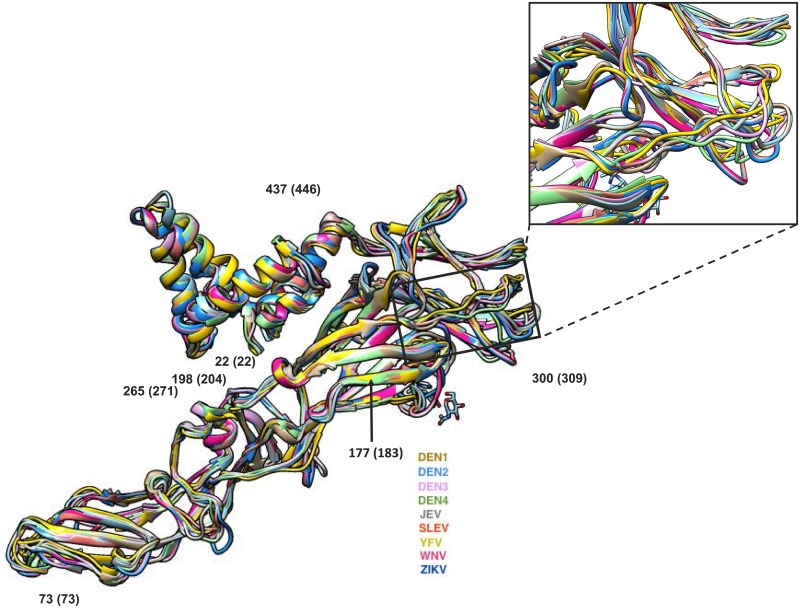
Comparison of predicted E glycoprotein structures for different Flavivirus taxa. Superimposed structural alignment of experimentally-derived and predicted E protein structures for 9 Flavivirus taxa. The black box indicates the region displayed in the inset panel. Inset shows a magnified view of a selected surface diagnostic 15-mer peptide that is located in a loop region in the E protein. DENV1, DENV2, DENV3, DENV4, JEV, SLEV, YFV, WNV, and ZIKV structures are colored brown, blue, light pink, green, gray, red, yellow, magenta, and navy blue, respectively. Structural regions displaying a single ribbon with multiple colors represent regions where no detectable structural variation is observed across taxa. In contrast, structural regions displaying multiple ribbons are those where the primary amino acid sequence is predicted to affect the 3D structure. Numbers in parentheses indicate the aligned amino acid position used throughout this study while numbers without parentheses represent the amino acid coordinate from the *in silico* structural alignment.

We next used three-dimensional protein structure modeling to determine whether the sequence divergence in diagnostic peptide regions would give rise to protein structural variability. To do so, we predicted mature E protein structures for seven of the ten Flaviviruses that currently lack such structures (DENV1, DENV3, DENV4, JEV, SLEV, WNV, YFV). These predicted structures, together with those existing for DENV2 and ZIKV mature E protein, were structurally aligned and had RMSD values below 3.0 indicating their close structural similarity (Fig 7). The model for ILHV was not included since it had an unexpectedly high root mean square deviation (RMSD) score between atomic positions and therefore did not pass our quality control criteria. Surprisingly, we found that although a large amount of diversity was observed in the amino acid sequences both across the whole protein as well as in the selected diagnostic peptide located in a major loop region (inset, Fig 7), this sequence diversity was not predicted to contribute to major structural variation. This analysis further confirms the validity of translating the surface accessibility scores from ZIKV E protein to all the other taxa and to project epitope regions across multiple taxa.

In summary, we report an analytical workflow to identify individual amino acid sites and 15-mer peptides that are significant, sensitive, and specific for distinguishing between multiple closely-related viruses. Applying this workflow to 10 different Flavivirus taxa revealed sets of viral peptide regions that are predicted to enable better post-convalescent antibody detection and diagnosis of mosquito-borne Flavivirus infection in humans.

## Discussion

In this work, we constructed a novel bioinformatics workflow that enabled the identification of residues that were specific to each of 10 mosquito-borne virus species and subtypes in the *Flavivirus* genus. This is especially important given the pressing need to develop diagnostics and detection methods with sufficient sensitivity and specificity to accurately differentiate between Flavivirus antigens across different virus species, such as ZIKV [38], and subtypes, such as DENV1-4 [48]. This workflow could easily be modified to predict unique peptide regions in other pathogens or to analyze nucleotide sequences in the context of generating reagents such as primers or probes for a variety of pathogens with high sequence similarity.

For each of the analyzed taxa we observed regions in the E or NS1 proteins that contained clusters of 15-mers with large numbers of diagnostic sites. Predicting which regions have adequate surface exposure adds additional characteristics for the identification of potential diagnostic peptide regions to serve as seroprevalence reagents. We also identified diagnostic sites that were buried within the folded protein structure, which could result in minor protein structure variations that alter molecular interactions. Additional wet-lab experimentation will be required to elucidate the contribution of these clusters.

We expected to see fewer diagnostic sites for ILHV due to the small amount of public sequence data available for this virus and because all of the available sequences are truncated. In contrast, both YFV and ZIKV have a relatively large number of diagnostic sites, presumably because they are more phylogenetically distant from the other mosquito-borne Flaviviruses. This phenomenon would lead to an increase of specific and sensitive diagnostic sites that were retained after each speciation event.

We have expanded on previous ZIKV-specific amino acid substitution analysis [49], including some that show the F279S and I311V substitutions are relevant for neutralizing antibody resistance [50]. Our analysis showed these positions differ between ZIKV and the 9 other Flavivirus taxa to some degree, but the average specificity and sensitivity of 51% and 82% (respectively) excluded them from being classified as diagnostic residues in our analysis. Similarly, the glycosylation site at N154 was not predicted as a diagnostic site because of its average specificity and sensitivity score of 94.7% due to the asparagine, the majority residue in ZIKV, being present at a sufficient frequency in the other Flavivirus taxa [51].

By combining the predicted diagnostic sites with surface accessibility data we have identified multiple regions that warrant follow-up with wet-lab experiments. Since the amino acid changes in the Flavivirus diagnostic peptide regions identified in this study are primarily on the outside surface of the E and NS1 proteins and result in only minor structural differences, we would largely expect surface accessibility and cross-reactive antibody binding to such regions to be maintained over time. Additionally, while even one amino acid change can affect antibody binding [52], the adaptive humoral response would still generate unique polyclonal antibodies capable of recognizing these differentiating regions between the various mosquito-borne Flaviviruses. These regions therefore warrant additional experimentation to determine those that could be incorporated into a species-specific diagnostic or detection method for these viruses.

For example, our results could be applied to the production of ZIKV-specific monoclonal antibodies by exposing an animal model to immunogenic peptides. Multiple injections of peptides containing a sufficient number of residues that were identified as being sensitive and specific for ZIKV should allow a large number of B-cells producing anti-viral antibodies to be collected for hybridoma and monoclonal antibody generation. We look forward to determining whether the surface-exposed diagnostic peptide regions in the E and NS1 proteins identified through this analysis overlap with the binding sites of existing and future ZIKV monoclonal antibodies that have reduced cross-reactivity [53].

Alternatively, synthetic peptides with multiple diagnostic sites could be used to detect and distinguish antibodies against these 10 Flaviviruses in human serum. Measuring antibody binding to sets of these viral diagnostic peptide regions would not require samples to be taken during acute infection to confirm past exposure to the pathogen and would consequently improve the accuracy of the incidence and prevalence rates being estimated for these viruses. Retrospective detection of anti-viral antibodies in serum using such peptides would take advantage of immunological memory and circulating antibodies to distinguish between past viral infections [54, 55]. Similarly, monitoring seroprevalence prospectively could track the emergence of new mosquito-borne Flavivirus outbreaks in at-risk regions and enable the timely implementation of appropriate preventative measures to minimize the number of new infections.

Given the severity of the current Zika virus outbreak, we are reporting and disseminating the results of this comparative analysis workflow to assist in the development of more accurate detection and diagnostic reagents. Deriving these results through the combination of robust bioinformatics methods should provide more reliable data for the development of better diagnostic and detection methods against mosquito-borne Flaviviruses.

## Conclusions

We established a novel bioinformatics workflow that enables the comprehensive identification of amino acid differences between groups of Flavivirus sequences. This analysis enabled the identification of sensitive and specific amino acid residues in the E and NS1 proteins that are capable of distinguishing between the 10 different mosquito-borne Flaviviruses infecting humans. Integrating data from three-dimensional protein structures revealed that a subset of these residues are exposed on the surface of these proteins and are therefore more likely to be recognized by species-specific host antibodies elicited during viral infection.

## Supporting information

S1 Table GenBank accession numbers and taxa assignments used to compare the protein sequences belonging to each taxon against the 9 other Flavivirus taxa. (XLSX)

S2 Table All diagnostic residues in the Flavivirus E protein for each taxon with their associated values. (XLSX)

S3 Table All diagnostic residues in the Flavivirus NS1 protein for each taxon with their associated values. (XLSX)

S4 Table All surface diagnostic E protein 15-mer peptides for each taxon with their amino acid positions and sequence. (XLS)

S5 Table All surface diagnostic NS1 protein 15-mer peptides for each taxon with their amino acid positions and sequence. (XLS)
